# Decompression retinopathy following nonpenetrating deep sclerectomy for primary congenital glaucoma

**DOI:** 10.1186/s12886-018-0906-z

**Published:** 2018-09-05

**Authors:** Prithvi Ramtohul, Maëva Chardavoine, Marie Beylerian, Aurore Aziz, Frédéric Matonti, Danièle Denis

**Affiliations:** 1 Centre Hospitalier Universitaire de l’Hôpital Nord, chemin des Bourrely, 13015 Marseille, France; 2 Centre Hospitalier Henri Duffaut d’Avignon, 305 rue Raoul Follereau, 84000 Avignon, France; 3 Aix en Provence, France

**Keywords:** Congenital glaucoma, Decompression retinopathy, Deep sclerectomy, Subhyaloid hemorrhage, Trabeculotomy, Vitrectomy

## Abstract

**Background:**

To describe a unique case of decompression retinopathy manifesting as pre-macular subhyaloid hemorrhage that occurs in a nine-day old child after undergoing a non-penetrating deep sclerectomy for primary congenital glaucoma.

**Case presentation:**

We report a single case of a 9-day-old boy who was referred to our department of ophthalmology for bilateral buphtalmia and corneal edema. He presented marked elevation of the intraocular pressure in both eyes (22 mmHg and 26 mmHg, in the right eye and left eye respectively) associated with significant optic nerve cupping.

Non-penetrating deep sclerectomy was performed for each eye, with effective reduction of the intraocular pressure during the first week postoperatively (11 mmHg and 7 mmHg in the right eye and left eye respectively). The right eye presented an isolated subhyaloid hemorrhage located in the pre-macular area, persisting 3 weeks after the initial surgery and requiring pars-plana vitrectomy to clear the visual axis. This uncommon complication was identified as decompression retinopathy.

The intraocular pressure remained controlled in the normal range three years after initial surgery in both eyes, with reversal of optic disc cupping.

**Conclusions:**

Decompression retinopathy is a potential complication after non-penetrating deep sclerectomy in primary congenital glaucoma, requiring prompt treatment strategy to prevent potential organic amblyopia.

## Background

Pediatric glaucoma is a condition characterized by an elevated intraocular pressure (IOP) and optic nerve damage, and it can be a potential cause of blindness [1]. It is classified as primary or secondary [2]. Primary glaucoma in childhood includes primary congenital glaucoma and juvenile-onset glaucoma, depending on the age of disease onset [3]. An isolated trabeculodysgenesis is involved in the pathogenesis of the primary glaucoma, resulting in an aqueous outflow impairment and elevated IOP [4]. Secondary pediatric glaucoma can either present at birth as a result of developmental ocular anomalies (nonacquired form) or develop later in childhood (acquired form) [5, 6].

Pediatric glaucoma is generally a surgical disease, because of the refractory nature of this condition in children, with medical treatment being used only as a temporary measure or in preparation for surgery [7].

Various surgical procedures have been described for the treatment of primary congenital glaucoma, including goniotomy [8], trabeculotomy [9], non-penetrating deep sclerectomy [9], trabeculectomy [10], and glaucoma drainage devices [11]. The use of wound-modulating agents, such as antimetabolites (5-flourouracil and mitomycin C), increase the success of filtering surgery in pediatric glaucoma [12]. Surgical strategy depends on the type of pediatric glaucoma, the timing of diagnosis and age, corneal clarity, degree of optic disc cupping, association with other ocular or systemic anomalies, and history of previous ocular surgery [13].

The type and severity of postoperative complications are various, depending on the surgical procedures [14]. Non-penetrating deep sclerectomy allows aqueous filtration from the anterior chamber to the subconjunctival space through a thin trabeculodescemetic membrane, avoiding the sudden IOP drop and thus lowering the incidence of postoperative complications associated with trabeculectomy [15].

Ocular decompression retinopathy was first described by Fechtner et al. as a complication of the abrupt iatrogenic lowering of intraocular pressure after glaucoma filtering procedure [16]. It is characterized by multiple foci of hemorrhages that may affect all layers of the retina. There are few published cases of decompression retinopathy and, to the best of our knowledge, there is only one reported case of decompression retinopathy occurring after non-penetrating deep sclerectomy [17].

We report a unique case of a nine-day-old boy who developed pre-macular subhyaloid hemorrhage as the only manifestation of a decompression retinopathy, after undergoing non-penetrating deep sclerectomy for primary congenital glaucoma.

## Case presentation

A 9-day-old Caucasian boy was referred to our department of ophthalmology for bilateral buphtalmia and corneal haze. He was born at term, by spontaneous delivery, of non-consanguineous parents, with no significant personal or family medical history, except a keratoconus in his older brother.

Clinical examination showed no dysmorphic facies, but bilateral megalocornea and buphtalmia, associated with photophobia. Right eye (OD) examination revealed nasal corneal clouding, deep anterior chamber and normal crystalline lens. Funduscopic examination confirmed optic disc cup (cup-to-disc ratio: 0.4) without any vitreous or retinal hemorrhages. Left eye (OS) examination disclosed an epiphora, associated with major corneal edema, deep anterior chamber and normal crystalline lens. Funduscopic examination showed optic disc cup (cup-to-disc ratio: 0.7). Detailed examination under inhalational anesthesia was performed urgently and demonstrated corneal asymmetry up to 1.5 mm (horizontal corneal diameter: 11.5 mm OD and 13 mm OS) and increased axial length in the left eye (axial length: 18.5 mm OD and 19.21 mm OS). The pachymetry showed central corneal thickness of 863 μm OD and 927 μm OS. The IOP (measured with Perkins MK2 tonometer, Haag-Streit, UK) was 22 mmHg OD and 26 mmHg OS. Gonioscopy showed an open angle with normal trabecular meshwork pigmentation in both eyes. Findings included the absence of angle recess and flat iris insertion. There was no peripheral anterior synechiae or embryotoxon (Table 1).

**Table 1:** Baseline characteristics and examination features

Patient Sex/Age/Ethnicity	Corneal diameters (mm)	IOP (mmHg)	Pachymetry (μm)	Axial length (mm)	Anterior chamber	Fundus examination
M/9 days/Caucasian	H OD: 11.5 V OD: 11	OD: 22	OD: 863	OD: 18.5	OD: No posterior embryotoxon or iridocorneal adhesion	OD: c/d 0.4 No vitreous or retinal hemorrhage
	H OS: 13 V OS: 12.5	OS: 26	OS: 927	OS: 19.21		
					OS: No posterior embryotoxon or iridocorneal adhesion	OS: c/d 0.7 No vitreous or retinal hemorrhage

Abbreviations: *c/d* cup-to-disc ratio, *H* horizontal, *OD* right eye: *OS* left eye, *V* vertical

A non-penetrating deep sclerectomy with trabeculotomy was firstly performed in the left eye under general anesthesia.

The surgical procedure started with a corneal traction suture temporally and nasally. This is followed by a conjunctival incision and localized tenectomy in the superior quadrant. A pentagonal scleral flap was created of 5 × 4 mm, half the scleral thickness, and dissection was carried forward 2 mm into clear cornea. We used mitomycin C, 0.4 mg/ml for two minutes, followed by careful irrigation. After this, a deep triangular scleral flap was marked, 4 × 4 × 2 mm, and dissection was performed forward until the level of Descemet’s membrane. The inner wall of Schlemm’s canal and the juxtacanalicular trabeculum were peeled off using toothed forceps (25-gauge Eckardt End-gripping Forceps, Dutch Ophthalmic, USA) without perforation of the trabeculodescemetic membrane. Then, a trabeculotomy was made, using the trabeculotome handpiece (Bausch & Lomb E0421 Harms Trabeculotome Set 1.8″ Left Right Ophthalmic, USA). The two third of the trabeculotome length were introduced into Schlemm’s canal to treat, nasally and temporally, an average of 70 degrees of the angle. A space maintainer (cylindrical collagen implant; Aquaflow®, Staar Surgical, Monrovia, CA, USA) was inserted in the scleral bed to prevent collapse of the superficial flap and secured with a single 10/0 Ethilon suture. Closure of the scleral flap and the conjunctiva was done by 10/0 Ethilon sutures and 10/0 Vicryl reabsorbable sutures respectively. The procedure was completed by subconjunctival injection of betamethasone (0,1 ml).

The same surgical protocol was performed in the right eye one week after. Postoperative treatment included antibiotic (tobramycin) and steroid (dexamethasone) eye drops, 6 times a day, during one week, followed by gradual reduction of the steroids over a period of 8 weeks and reduction of antibiotic dose.

**Fig. 1:**
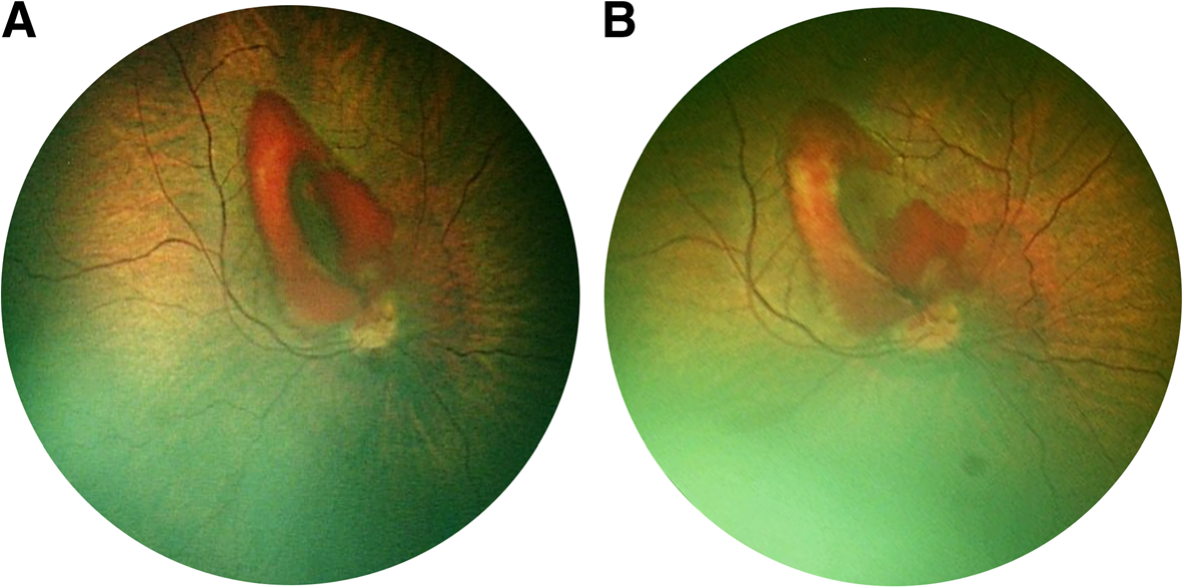
Postoperative fundus photography at one week (**a**) and three weeks (**b**), using the RetCam 3 (Clarity Medical Systems, Pleasanton, California, USA). It showed an incomplete resolution of the subhyaloid hemorrhage, still occluding the visual axis

One week postoperatively, the IOP was 11 mmHg OD and 7 mmHg OS. The anterior chamber and the bleb were well-formed in both eyes (Table 2). Fundoscopy exhibited a well-circumscribed, crab claw-shaped hemorrhage, corresponding to a pre-macular subhyaloid hemorrhage in the right eye, resulting in an artificially decrease of the OD axial length (Fig. 1). Fundus examination of the left eye was unremarkable. Complete blood coagulation analysis was performed and revealed no significant anomaly, excepted moderate polycythemia (hemoglobin: 22.8 g/dL). The patient had no incident of coughing or sneezing postoperatively. A suspected diagnosis of decompression retinopathy was proposed. Close observation was firstly decided. Three weeks after initial surgery, as there was no improvement in subhyaloid hemorrhage, pars plana vitrectomy (25-gauge Stellaris PC System, Bausch & Lomb, Rochester, NY, USA) of the right eye was performed, allowing a complete resolution without any recurrence.

**Table 2:** Postoperative examination features at one week, three weeks and three years

Date of control	Corneal diameters (mm)	IOP (mmHg)	Pachymetry (μm)	Axial length (mm)	Anterior chamber	Fundus examination	Medical decision
One week	H OD: 11 V OD: 10	OD: 11	OD: 605	OD: 17.64	OD: Well-formed bleb and AC	OD: Subhyaloid hemorrhage, c/d 0.3	OD: Close observation
	H OS: 12.5 V OS: 11.5	OS: 7	OS: 871	OS: 19.37	OS: Well-formed bleb and AC	OS: c/d 0.6	
Three weeks	H OD: 10 V OD: 10	OD: 13	OD: 561	OD: 17.02	OD: Clear cornea, well-formed bleb and AC	OD: Persisting subhyaloid hemorrhage, c/d 0.1	OD: Vitrectomy
	H OS: 12.5 V OS: 11.5	OS: 22	OS: 870	OS: 18.71	OS: Decrease cornea edema, well-formed bleb and AC	OS: c/d 0.5	OS: 5-FU bleb needling
Three years	H OD: 12 V OD: 11	OD: 8	OD: 589	OD: 23.93	OD: Flat bleb, no cataract	OD: c/d 0.1	Refractive correction: OD: −6.25 (−1.50; 175°)
	H OS: 12.5 V OS: 12	OS: 9	OS: 612	OS: 21.72	OS: Flat bleb, no cataract	OS: c/d 0.1	OG: + 1.50 (−2.00; 170°)

Abbreviations: *AC* anterior chamber, *c/d* cup-to-disc ratio, *H* horizontal, *OD* right eye: *OS* left eye, *V* vertical, *5-FU* 5-fluorouracil

Three years after initial surgery, the IOP remained controlled in the normal range for both eyes, with reversal of optic disc cupping (c/d 0.1 OD and c/d 0.1 OS). A relevant finding was the onset of high myopia in the OD requiring refractive correction (Table 2).

## Discussion and conclusions

Neonatal retinal and vitreous hemorrhages are mostly influenced by the modalities of delivery [18]. In fact, caesarean delivery is associated with a higher incidence of retinal and vitreous hemorrhages. Other main parameters correlate with retinal and vitreous hemorrhages are gestational age, birth weight, asphyxia, scalp hematoma and precipitate labour [19]. Newborn retinal hemorrhages are commonly bilateral, predominantly intraretinal and in the posterior pole [20]. In our case, the patient was born by eutocic vaginal delivery and presented no reported risk factor.

Other etiologies associated with vitreous hemorrhages in pediatric age group are separated in traumatic and nontraumatic causes. Nontraumatic causes are, in order of frequency, retinoblastoma, Terson’s syndrome, persistent fetal vasculature, idiopathic, regressed retinopathy of prematurity, familial exudative vitreoretinopathy, intermediate uveitis, associated with lymphoblastic leukemia, retinitis pigmentosa, familial retinal artery macroaneuvrysm, nanophtalmos, neonatal meningitis, panuveitis (brucellosis), Stickler diseases with rhegmatogenous retinal detachment (RRD), arteriovenous malformation, Coats disease and Marfan syndrome with RRD [21]. Coagulation disorders could also be implied, including leukemia, haemophilia, Von Willebrand disease and Protein C deficiency [22]. In our case, an isolated transient polycythemia was found, and no recent traumatic history or vitreoretinal diseases were associated.

After excluding an inherent disease, a postoperative complication of the non-penetrating deep sclerectomy was suspected. Common complications of non-penetrating deep sclerectomy include transient hyphema, shallows and flats in the anterior chamber, hypotonia or hypertonia, bleb fibrosis, and more rarely, choroidal effusion, detachment or hemorrhage, macular edema and decompression retinopathy [23].

We hypothesized that the underlying mechanism involves a decompression retinopathy. In fact, post-operative abrupt hypotonia leads to rapid increase in central retinal artery flow, increasing retinal capillary perfusion pressure beyond its autoregulatory capacity, resulting in hemorrhages [24]. Thus, the extravasated blood may sediment under the posterior hyaloid membrane [25]. The main differential diagnosis for decompression retinopathy includes venous occlusion and Valsalva’s retinopathy. Venous occlusion was excluded due to the lack of venous dilatation, peripheral retinal hemorrhages and optic disc edema. Similarly, the possibility of Valsalva’s retinopathy was remote because the patient had no incident of coughing or sneezing postoperatively.

The incidence of decompression retinopathy in children has been evaluated at 5.2% in a recent study [26], slightly higher than the 3.0% reported by Jung et al. in adult population. According to the current vascular and mechanical theories for the pathophysiology of decompression retinopathy, the lower degree of resistance in the child sclera may explain this higher incidence. Therefore, mechanical deformation of the posterior segment structures, including posterior cortical vitreous, retinal capillaries and lamina cribrosa might be intensified [27].

In pediatric population, the specific high density of the collagen fibrils meshwork with interspersed extensive arrays of long hyaluronan molecules explains the slower clearance of vitreous hemorrhage, compared with adults [28]. Miller-Mecks et al. demonstrated that visual axis obstruction induced by vitreous hemorrhage may engender high myopia [29], persisting after vitreous clarity recovery, which contributes to the onset of an amblyopia [30]. Our case report is in agreement with Miller-Mecks observation, as high myopia developed in the right eye requiring refractive correction. It is a challenge to determine until when vitrectomy can be delayed. Ferrone et al. suggested that vitreous hemorrhage sequelae are potential from five weeks, supporting a prompt treatment strategy [31]. In our case, pars plana vitrectomy was performed at the age of 4 weeks. Given the surgical difficulty and the extremely high risk of serious postoperative complications, such procedure should not be generalised.

To minimize the risk of decompression retinopathy, several measures should be considered: optimized control of the IOP before glaucoma surgery, performing a paracentesis to allow slow drainage of aqueous humor, use of viscoelastic material in anterior chamber to allow progressive lowering of IOP, and tight and releasable sutures of the scleral flap to prevent marked and prolonged hypotonia [26].

Non-penetrating deep sclerectomy is an effective procedure in congenital glaucoma and is considered theoretically safer than trabeculectomy [32]. In this case report, we described a child with primary congenital glaucoma who developed pre-macular subhyaloid hemorrhage after non-penetrating deep sclerectomy, as the only manifestation of a decompression retinopathy. To our knowledge, this is the first report of such complication occurring after non-penetrating deep sclerectomy for primary congenital glaucoma and requiring vitrectomy. This uncommon complication needs to be considered as it is a potential cause of organic amblyopia.

### Patient consent

The patient could not give consent. The parents gave verbal and written permission. This report does not contain any personal information that could lead to the identification of the patient.
